# Risk of Parkinson’s disease in patients with schizophrenia: Impact of antipsychotic medication use

**DOI:** 10.1371/journal.pone.0346233

**Published:** 2026-05-04

**Authors:** Jin Yeon Gil, Kyung Hyun Min, Jun Hyeob Kim, Jun Hyuk Park, Ji Min Han, Kyung Eun Lee

**Affiliations:** College of Pharmacy, Chungbuk National University, Cheongju-si, South Korea; NYU Grossman School of Medicine: New York University School of Medicine, UNITED STATES OF AMERICA

## Abstract

**Background/objectives:**

Parkinson's disease (PD) is a progressive neurodegenerative disorder characterized by motor and non-motor symptoms, and its incidence is steadily increasing. Schizophrenia (SCZ) is a severe psychiatric disorder involving both positive and negative symptoms. Both diseases share dopaminergic dysregulation, but the relationship between schizophrenia and PD and the role of antipsychotic treatment in the risk of PD are not well understood. This study aimed to explore the risk of PD in individuals with schizophrenia and assess the influence of antipsychotic medications on this risk.

**Methods:**

This retrospective cohort study used a customized database from the National Health Insurance Service (NHIS) of South Korea, which included patients diagnosed with schizophrenia and matched controls. We performed propensity score matching and adjusted for various demographic, clinical, and comorbidity variables. Competing risk analysis and Cox regression were used to analyze the relationship between schizophrenia and PD, considering both antipsychotic medication exposure and comorbid conditions.

**Results:**

We found that individuals with schizophrenia had a significantly higher risk of developing PD than the control subjects, with an adjusted hazard ratio of 3.198. Among schizophrenia patients, compared with control, risperidone use was associated with an increased risk of PD, whereas quetiapine use was associated with a reduced risk. Significant comorbidities, such as head injury and cerebrovascular disease, also contributed to the increased risk of PD.

**Conclusions:**

Our study highlights the elevated risk of PD in individuals with schizophrenia, emphasizing the need for close monitoring of this patient population. The impact of antipsychotic medications, particularly risperidone and quetiapine, on the development of PD warrants further investigation to better understand the long-term effects of these treatments.

## Introduction

Parkinson’s disease (PD) is the second most common neurodegenerative disorder, and its incidence has markedly increased over the past 20 years [1]. PD is more prevalent in men than in women [2], and the risk of onset is highest in individuals aged between 60 and 80 [3]. PD is characterized by both motor and non-motor symptoms; typical motor symptoms include bradykinesia, rigidity, and tremor during early stages, and posture alterations and freezing of gait becoming prominent in later stages [4–5]. Although the exact etiology of PD remains unclear, both environmental and genetic factors are believed to contribute to its development. Smoking (nicotine) and caffeine have been well established as having potential protective effects against the development of Parkinson’s disease (PD) [4,6–9].

PD has also been linked to psychiatric conditions such as depression and anxiety, with some studies suggesting an elevated risk of PD in individuals with pre-existing psychiatric disorders [10–14].

Schizophrenia (SCZ), a complex psychiatric disorder, presents a wide range of symptoms, although its precise cause remains unknown [15]. Schizophrenia is characterized by positive and negative symptoms. Positive symptoms include hallucinations and paranoid delusions, while negative symptoms involve emotional blunting, loss of motivation, and decreased willpower [16].

Second-generation (atypical) antipsychotics (SGAs), except clozapine, are used as first-line monotherapy for schizophrenia. If the patient shows little or no response, the treatment should progress to stage 2, which involves monotherapy with either a different SGA or a first-generation (typical) antipsychotic (FGA) [17]. SGAs, such as risperidone and olanzapine, act primarily by blocking serotonin (5-HT2A) and dopamine (D2) receptors, which are believed to help alleviate both positive and negative symptoms [18–19]. In contrast, FGAs, such as haloperidol, primarily block dopamine D2 receptors, and their use is often accompanied by higher risks of extrapyramidal side effects [20]. Both PD and schizophrenia are biologically linked to dopamine dysregulation in the brain [21]. PD treatment involves medications such as levodopa, which increase dopamine levels, whereas schizophrenia treatment uses dopamine antagonists, like SGAs and FGAs, to reduce dopamine activity, highlighting their opposing neurochemical mechanisms. Sharing this biological pathway between PD and schizophrenia necessitates careful analysis to exclude confounding drug effects when investigating the relationship between the two disorders.

Previous research has suggested that individuals with psychiatric disorders or psychotic symptoms may have an elevated risk of developing PD [22–23]. A study that explored PD risk factors has reported a notably high odds ratio of 4.48 for schizophrenia [24]. However, most previous studies have focused primarily on psychiatric disorders or psychotic symptoms as a whole, with limited attention to the specific association between schizophrenia and PD. To date, only one study has examined this relationship, but it did not account for the potential modifying effects of antipsychotic medications [25]. Therefore, we conducted a retrospective cohort study to evaluate the risk of PD associated with schizophrenia and the influence of antipsychotic medications prescribed at the time of schizophrenia diagnosis on PD development.

## Materials and methods

### Database and study population

We conducted a retrospective cohort study using a customized database provided by the National Health Insurance Service (NHIS) of South Korea. Parkinson’s disease (PD) is included in the NHIS registration program for intractable diseases, requiring physician-certified diagnoses based on standardized criteria provided by the NHIS [26]. This database includes demographic characteristics, health insurance claim codes (procedures and prescriptions), data from the International Classification of Diseases, 10th Revision (ICD-10), health examination data, and socioeconomic data (residence and income) of the study subject in South Korea from 2010 to 2021. The data used in this study were accessed for research purposes on July 11, 2022. The data that support the findings of this study are available from the National Health Insurance Sharing Service of South Korea, but restrictions apply to their availability due to the regulations that prohibit the public release of individual-level data.

Patients whose records were maintained from 2010 to 2021 were selected. Schizophrenia was defined according to the ICD-10 code (F2*), with patients having one inpatient record or two outpatient records within one year. To define incident new cases, patients diagnosed with schizophrenia in 2010 were excluded. Additionally, patients under 18 years of age were excluded.

Patients with schizophrenia were matched with patients without schizophrenia using the propensity score matching method in a 1:2 ratio on the basis of the Charlson Comorbidity Index (CCI) score, age, gender, and index year; the CCI score was calculated from records one year prior to the index date. We used a caliper width of 0.2 standard deviations of the logit of the propensity score to ensure close matching between schizophrenia and control subjects, with a standardized mean difference of ≥ 0.1 indicating imbalance. For patients without schizophrenia, the index year was randomly assigned between January 1, 2011, and the end date. The end date was defined as the date of PD diagnosis, death, or the last day of data collection (December 31, 2021).

### Covariate

The inclusion of covariates potentially associated with PD was based on previous studies. These covariates included demographic factors such as sex, age, and index year, as well as a range of clinical conditions: gastrointestinal diseases, osteoporosis, inflammatory arthritis, head injury, chronic obstructive pulmonary disease (COPD), type 2 diabetes, hyperlipidemia, hypertensive disease, end-stage renal disease, coronary heart disease, and cerebrovascular disease [6,24,27–34]. To account for the overall comorbidity burden after matching, the CCI was also included as a covariate. To control for the potential effects of mental disorders and antipsychotic medications on PD, mental disorders (ICD-10, F00-F69) and related medications (ATC, N05A–N06D) were also included as covariates [23,35,36]. Covariates were based on records from one year prior to the outcome.

### Outcome

The outcome was incident PD, defined as a new diagnosis of PD using the ICD-10 code G20 occurring at least one year after the initial schizophrenia diagnosis. To minimize diagnostic errors, PD was defined as at least one inpatient diagnosis or two outpatient diagnoses with the G20 code within a one-year period.

### Statistical methods

To compare characteristics between the patients with schizophrenia and control groups, t-tests were used for continuous variables, and chi-squared tests were used for binary variables. The phi correlation coefficient was used to measure the association between two binary variables. Cox regression analysis was used to calculate the crude and adjusted hazard ratios with 95% confidence intervals (CI) of PD incidence rates in the two groups. Survival analysis was performed using the Kaplan–Meier method, with significance based on the log-rank test. Fine–Gray’s competing risk analysis was used to determine the risk of PD, considering death as a competing risk factor [37]. We also investigated the relationship between the most commonly prescribed antipsychotic medications for patients with schizophrenia and the development of PD. Using the intent-to-treat method, we performed Cox regression, Kaplan–Meier analysis, and log-rank tests based on the medications prescribed at the time of the initial diagnosis. Sensitivity analysis was conducted with 1, 2, or 3 years before the end date, due to the limited knowledge about the onset of PD disease in schizophrenia patients.

All statistical analyses were performed using SAS Enterprise Guide version 9.4 (SAS Institute Inc., Cary, NC, USA), with a two-sided CI of 0.05 considered to indicate statistical significance.

### Ethics statement

The study was conducted in accordance with the Declaration of Helsinki and was approved by the Institutional Review Board of Chungbuk National University (CBNU-202204-HR-0068; approval date: 18 April 2022). This study utilized de-identified secondary data provided by the National Health Insurance Service (NHIS); therefore, the requirement for individual informed consent was waived. To ensure data security and privacy, all analyses were conducted within the NHIS-controlled secure research environment with restricted access. Access to the data was available only through the designated NHIS analysis center, and remote access from personal computers or external networks was not permitted. Only NHIS-approved researchers were authorized to enter the analysis center and access the data in accordance with NHIS data governance policies.

## Results

### Baseline characteristics and univariate analysis

**Fig 1:**
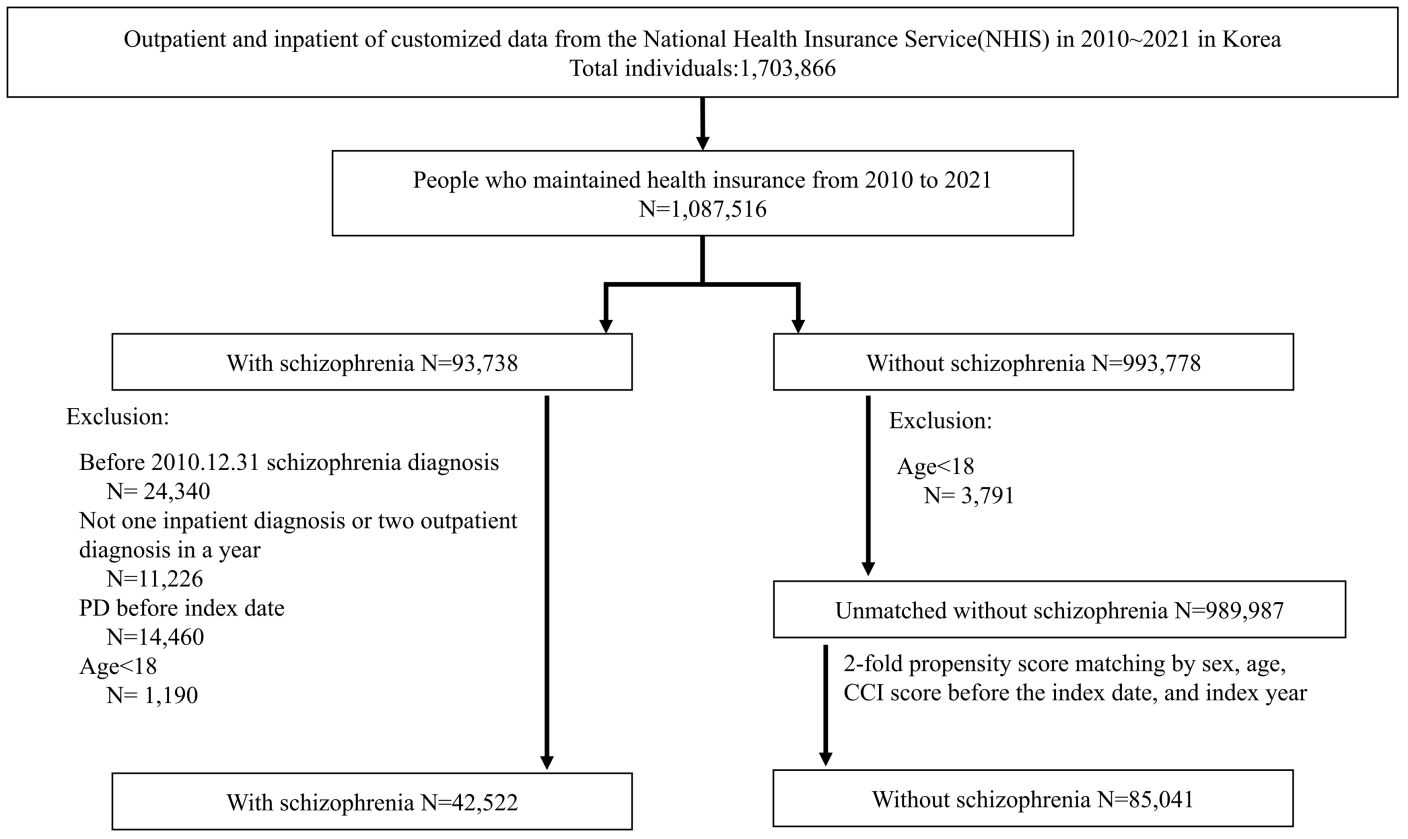
Study flow chart.

A total of 42,522 patients with schizophrenia were matched to 85,041 controls by age, gender, matching-period CCI score, and index year (Fig 1). The average age of the patients with schizophrenia and the control group were 66.26 ± 18.48 years and 66.36 ± 18.27 years, respectively. Both groups had a higher proportion of females than males (60.6%), and 53% of patients in each group scored three or more points on the matching-period CCI.

Univariate analysis was conducted to examine the associations between schizophrenia status and various variables. The patients with schizophrenia had a significantly higher prevalence of comorbidities than did the control patients; the comorbidities included various mental and behavioral disorders, head injuries, COPD, and cerebrovascular disease. In addition, a higher number of users was observed across all medications among patients with schizophrenia compared to the control group. Specifically, the usage rate of N05A was approximately 51% higher among patients with schizophrenia than in the control group (Table 1).

**Table 1:** Baseline characteristics of patients with schizophrenia and control participants.

	Control group N = 85041 (%)	Patients with SCZ N = 42522 (%)	P-value	SMD^*^
Sex			0.99	
Male	33504 (39.40)	16753 (39.40)		<0.001
Female	51537 (60.60)	25769 (60.60)		<0.001
Age (mean ± SD)	66.36 ± 18.27	66.26 ± 18.48	0.40	0.005
Index year (mean ± SD)	2016.2 ± 3.12	2016.2 ± 3.11	0.07	<0.001
Charlson Comorbidity Index (Post-Matching)			<0.001	
0	20039 (23.56)	11554 (27.17)		0.083
1-2	26600 (31.28)	12674 (29.81)		0.031
3≤	38402(45.16)	18294 (43.02)		0.043
Duration, year (mean±SD)	4.03 ± 2.98	2.85 ± 2.77	<0.001	0.410
Comorbidities				
Organic, including symptomatic, mental disorders	17378 (20.43)	21699 (51.03)	<0.001	0.673
Mental and behavioral disorders due to psychoactive substance use	396 (0.47)	2211 (5.20)	<0.001	1.082
Mood (affective) disorders	15290 (17.98)	26433 (62.16)	<0.001	1.009
Neurotic, stress-related and somatoform disorders	19299 (22.69)	18873 (44.38)	<0.001	0.472
Behavioral syndromes associated with physiological disturbances and physical factors	6584 (7.74)	10918 (25.68)	<0.001	0.495
Disorders of adult personality and behavior	77 (0.09)	478 (1.12)	<0.001	0.133
Gastric disease	68038 (80.01)	29403 (69.15)	<0.001	0.251
Osteoarthritis	31856 (37.46)	12445 (29.27)	<0.001	0.174
Inflammatory arthritis	4519 (5.31)	1995 (4.69)	<0.001	0.028
Head injury	1600 (1.88)	1671 (3.93)	<0.001	0.122
Chronic Obstructive Pulmonary Disease (COPD)	3711 (4.36)	2096 (4.93)	<0.001	0.027
Diabetes mellitus (DM)	29678 (34.90)	12727 (29.93)	<0.001	0.106
Hyperlipidemia	47089 (55.37)	19468 (45.78)	<0.001	0.192
Hypertensive disease	50049 (58.85)	21938 (51.59)	<0.001	0.146
End-stage renal disease (ESRD)	4481 (5.27)	2126 (5.00)	0.040	0.012
Coronary heart disease	14820 (17.43)	7014 (16.49)	<0.001	0.025
Cerebrovascular disease	16462 (19.36)	10475 (24.63)	<0.001	0.127
Medicine, ATC code				
N05A (Antipsychotics)	16636 (19.56)	30243 (71.12)	<0.001	1.210
N05B (Anxiolytics)	26742 (31.45)	23560 (55.41)	<0.001	0.498
N05C (Hypnotics and sedatives)	15477 (18.20)	13134 (30.89)	<0.001	0.298
N06A (Antidepressants)	14193 (16.69)	19822 (46.62)	<0.001	0.679
N06B (Psychostimulants for ADHD)	3775 (4.44)	3404 (8.01)	<0.001	0.148
N06D (Anti-dementia drugs)	8172 (9.61)	16040 (37.72)	<0.001	0.700

*Standardized Mean Difference.

### Collinearity and multivariable analysis of risk factors

To ensure the reliability of multivariable analysis, we examined collinearity. Variables with a phi coefficient of 0.4 or higher were excluded, including CCI score (from the same period as the comorbidities after matching), medication (N05A–N06D), mood (affective) disorders, and hyperlipidemia [38]. When high collinearity was observed between two diseases, the disease with more associations with other variables was excluded. In cases of collinearity between a disease and a medication, the medication was excluded.

After excluding collinear variables and adjusting for the remaining factors, competing risk analysis revealed that schizophrenia was associated with a 3.198-fold increased risk of PD (95% CI = 3.126–3.217). The risk of developing PD was higher in males than in females (aHR = 1.103; CI = 1.082–1.126). Several comorbidities have been associated with an increased risk of Parkinson’s disease, including neurotic, stress-related, and somatoform disorders, cerebrovascular disease, head injury, behavioral syndromes associated with physiological disturbances and osteoarthritis (Table 2).

**Table 2:** Crude and adjusted hazard ratio (HR) for parkinson’s disease using cox proportional regression and fine-gray competing risk analysis.

Variable	No competing risk analysis				Competing risk analysis^†^	
	Crude HR(95% CI)	P-value	Adjusted HR (95% CI)	P-value	Adjusted HR (95% CI)	P-value
Schizophrenia	3.845 (3.772-3.920)	<0.001	3.310 (3.238-3.384)	<0.001	3.198 (3.126-3.217)	<0.001
Sex						
Male	1.201 (1.178-1.224)	<0.001	1.121 (1.099-1.143)	<0.001	1.103 (1.082-1.126)	<0.001
Female	1.00 (reference)		1.00 (reference)		1.00 (reference)	
Age	0.989 (0.988-0.989)	<0.001	0.990 (0.989-0.990)	<0.001	0.986 (0.986-0.987)	<0.001
Charlson Comorbidity Index^#*^						
0	1.00 (reference)		–	–	–	–
1-2	0.756 (0.737-0.776)	<0.001	–	–	–	–
3	0.833 (0.814-0.852)	<0.001	–	–	–	–
Comorbidities						
Organic, including symptomatic, mental disorders	1.409 (1.381-1.437)	<0.001	0.993 (0.970-1.017)	0.58	0.980 (0.957-1.004)	0.11
Mental and behavioral disorders due to psychoactive substance use	2.400 (2.290-2.515)	<0.001	1.011 (0.963-1.061)	0.66	1.021 (0.977-1.068)	0.36
Mood (affective) disorders*	2.500 (2.453-2.548)	<0.001	–	–	–	–
Neurotic, stress-related and somatoform disorders	1.991 (1.954-2.030)	<0.001	1.508 (1.476-1.541)	<0.001	1.521 (1.489-1.554)	<0.001
Behavioral syndromes associated with physiological disturbances and physical factors	1.938 (1.894-1.982)	<0.001	1.112 (1.084-1.140)	<0.001	1.122 (1.095-1.149)	<0.001
Disorders of adult personality and behavior	2.738 (2.487-3.013)	<0.001	1.038 (0.942-1.144)	0.45	1.008 (0.923-1.100)	0.86
Gastric disease	0.661 (0.647-0.675)	<0.001	0.689 (0.673-0.706)	<0.001	0.702 (0.686-0.719)	<0.001
Osteoarthritis	0.834 (0.817-0.851)	<0.001	1.042 (1.019-1.066)	0.01	1.078 (1.055-1.102)	<0.001
Inflammatory arthritis	0.966 (0.926-1.008)	0.11	1.029 (0.985-1.074)	0.20	1.024 (0.982-1.068)	0.26
Head injury	1.640 (1.561-1.723)	<0.001	1.249 (1.188-1.312)	<0.001	1.228 (1.168-1.290)	<0.001
COPD	0.985 (0.941-1.031)	0.52	0.981 (0.936-1.028)	0.42	0.949 (0.906-0.994)	0.03
DM	0.807 (0.790-0.824)	<0.001	0.925 (0.904-0.946)	<0.001	0.937 (0.916-0.957)	<0.001
Hyperlipidemia*	0.711 (0.695-0.724)	<0.001	–	–	–	–
Hypertensive disease	0.659 (0.647-0.672)	<0.001	0.785 (0.767-0.803)	<0.001	0.800 (0.782-0.819)	<0.001
ESRD	0.792 (0.756-0.830)	<0.001	0.887 (0.845-0.931)	<0.001	0.840 (0.801-0.881)	<0.001
Coronary heart disease	0.874 (0.851-0.897)	<0.001	0.983 (0.956-1.011)	0.23	0.975 (0.948-1.002)	0.07
Cerebrovascular disease	1.307 (1.279-1.336)	<0.001	1.346 (1.314-1.379)	<0.001	1.346 (1.314-1.379)	<0.001
Medicine						
N05A (Antipsychotics)^*^	2.744 (2.692-2.798)	<0.001	–	–	–	–
N05B (Anxiolytics)^*^	2.039 (2.000-2.078)	<0.001	–	–	–	–
N05C (Hypnotics and sedatives)^*^	1.473 (1.443-1.504)	<0.001	–	–	–	–
N06A (Antidepressants)^*^	2.102 (2.062-2.143)	<0.001	–	–	–	–
N06B (Psychostimulants for ADHD)^*^	1.958 (1.895-2.023)	<0.001	–	–	–	–
N06D (Anti-dementia drugs)^*^		<0.001	–	–	–	–

† Fine-gray comepeting risk analysis.

* Variables with a Phi correlation coefficient greater than 0.4 were excluded from the adjusted HR analysis.

# In the same period as the comorbidities after matching.

### Sensitivity analysis and Kaplan-Meier survival

**Fig 2:**
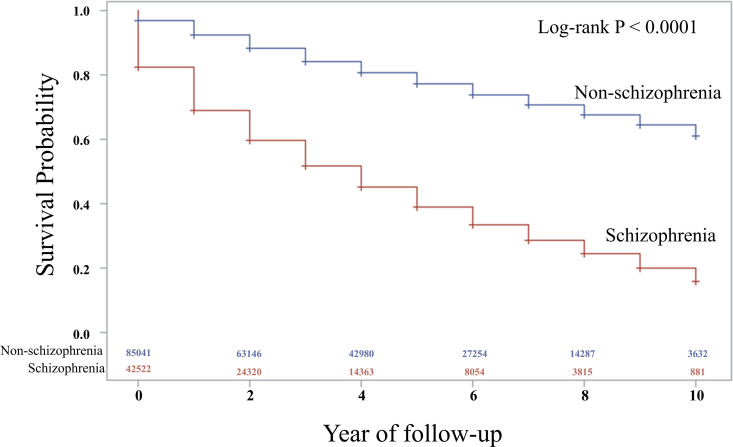
Kaplan-Meier Plot Showing Event-Free Survival for Parkinson’s Disease by Schizophrenia.

Sensitivity analysis, which included competing risk analysis, demonstrated that the aHR for PD remained significant across 1-, 2-, and 3-year periods of comorbidities, confirming the robustness of the findings (Table 3). A Kaplan–Meier plot of PD-free survival rates during the follow-up period revealed a significantly steeper decline in survival for patients with schizophrenia than for controls, particularly over the 10-year follow-up period. By the end of 10 years, the cumulative PD-free survival rate for patients with schizophrenia was significantly lower than that of the control group, highlighting the substantial long-term impact of schizophrenia on the risk of PD (log-rank test; P < 0.0001) (Fig 2).

**Table 3:** Sensitivity Analysis of PD Risk by Exposure Duration: Fine-Gray and Cox Models.

Covariate	No competing risk model		Fine-gray competing model	
duration	Adjusted HR (95% CI)	P-value	Adjusted HR (95% CI)	P-value
1 years	3.310 (3.238-3.384)	<0.001	3.198 (3.126-3.217)	<0.001
2 years	3.289 (3.217-3.363)	<0.001	3.147 (3.075-3.221)	<0.001
3 years	3.276 (3.204-3.351)	<0.001	3.118 (3.045-3.193)	<0.001

### Impact of antipsychotic medications

**Fig 3:**
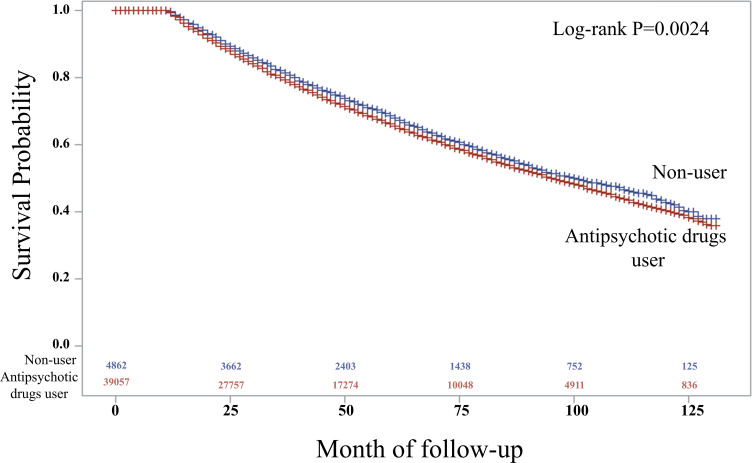
Event-Free Survival in Parkinson’s Disease: Kaplan-Meier Analysis by antipsychotic drugs.

At the initial diagnosis, antipsychotic drugs (N05A) were most frequently prescribed to schizophrenia patients (S1 fig). In the multivariate analysis, the use of N05A drugs was significantly associated with the development of PD (HR: 1.076, 95% CI: 1.023–1.132, p = 0.0045). Among comorbidities, neurotic, stress-related, and somatoform disorders showed the highest association (HR: 1.380, 95% CI: 1.333–1.429, p < 0.001), followed by mental and behavioral disorders due to psychoactive substance use (HR: 1.258, 95% CI: 1.184–1.337, p < 0.001) (Table 4). Kaplan–Meier plots indicated that PD-free survival rates were significantly lower in patients prescribed antipsychotics than in non-users (Fig 3).

**Table 4:** Adjusted HR for PD in antipsychotic drugs prescription patients.

Variable	Adjusted HR (95% CI)	P-value
Age	0.983 (0.982-0.984)	<0.001
Antipsychotic drugs	1.076 (1.023-1.132)	0.01
Sex	1.046 (1.011-1.082)	0.01
Organic, including symptomatic, mental disorders	1.177 (1.132-1.223)	<0.001
Mental and behavioral disorders due to psychoactive substance use	1.258 (1.184-1.337)	<0.001
Neurotic, stress-related and somatoform disorders	1.380 (1.333-1.429)	<0.001
Behavioral syndromes associated with physiological disturbances and physical factors	1.241 (1.198-1.286)	<0.001
Disorders of adult personality and behavior	1.171 (1.044-1.314)	0.01
Gastric disease	1.092 (1.045-1.141)	<0.001
Osteoarthritis	1.056 (1.017-1.096)	0.01
Inflammatory arthritis	1.016 (0.948-1.089)	0.65
Head injury	1.233 (1.148-1.325)	<0.001
Chronic Obstructive Pulmonary Disease	0.979 (0.909-1.055)	0.58
Diabetes mellitus	0.919 (0.885-0.954)	<0.001
Hypertensive disease	0.922 (0.888-0.958)	<0.001
End-stage renal disease	0.785 (0.725-0.851)	<0.001
Coronary heart disease	0.958 (0.914-1.003)	0.07
Cerebrovascular disease	1.118 (1.074-1.163)	<0.001

**Fig 4:**
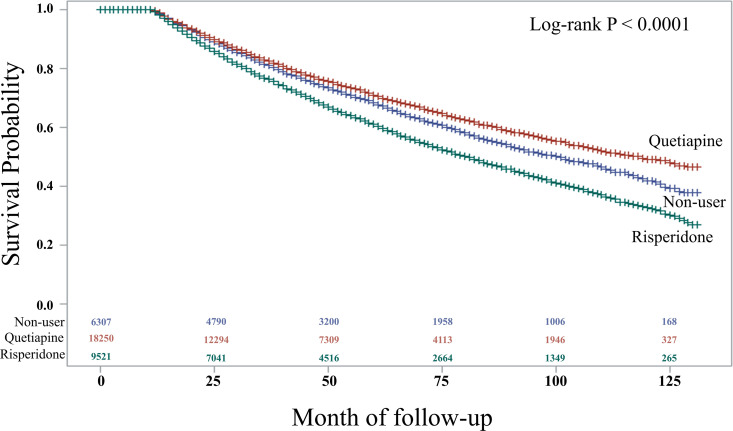
Kaplan-Meier Curves for Event-Free Survival in Parkinson’s Disease: Quetiapine, Risperidone, and Non-Users.

Analysis of medication types revealed that quetiapine was the most commonly prescribed drug (20,485 patients), followed by risperidone (9,521 patients) and aripiprazole (5,052 patients)(S2 fig). In the subgroup multivariate analysis, relative to non-users risperidone use was significantly associated with an increased risk of PD (HR: 1.203, 95% CI: 1.142–1.267, p < 0.001), whereas quetiapine use was associated with a reduced risk (HR: 0.946, 95% CI: 0.899–0.995, p = 0.0317). Furthermore, aripiprazole use was also significantly associated with an increased risk of PD compared with non-users in a separate analysis (HR: 1.160, 95% CI: 1.103–1.221, p < 0.001). Among comorbidities, mental and behavioral disorders due to psychoactive substance use (HR: 1.305, 95% CI: 1.218–1.398, p < 0.001) and neurotic, stress-related, and somatoform disorders (HR: 1.338, 95% CI: 1.286–1.393, p < 0.001) showed the strongest associations (Table 5). Kaplan–Meier analysis revealed that quetiapine users had a lower risk of developing PD, whereas risperidone users had a higher risk than non-users. Accordingly, the PD-free survival was significantly longer in quetiapine users and was significantly shorter in risperidone users than the non-users (Fig 4).

**Table 5:** Adjusted HR for PD in quetiapine, risperidone prescription patients.

Variable	Hazard Ratio (95% CI)	P-value
Age	0.984 (0.983-0.985)	<0.001
Exposure		
Quetiapine	0.946 (0.899-0.995)	0.03
Risperidone	1.203 (1.142-1.267)	<0.001
Sex	1.049 (1.008-1.092)	0.02
Organic, including symptomatic, mental disorders	1.177 (1.125-1.232)	<0.001
Mental and behavioral disorders due to psychoactive substance use	1.305 (1.218-1.398)	<0.001
Neurotic, stress-related and somatoform disorders	1.338 (1.286-1.393)	<0.001
Behavioral syndromes associated with physiological disturbances and physical factors	1.246 (1.196-1.299)	<0.001
Disorders of adult personality and behavior	1.170 (1.021-1.340)	0.02
Gastric disease	1.116 (1.061-1.174)	<0.001
Osteoarthritis	1.051 (1.007-1.097)	0.02
Inflammatory arthritis	1.081 (0.997-1.173)	0.06
Head injury	1.250 (1.153-1.354)	<0.001
Chronic Obstructive Pulmonary Disease	0.984 (0.904-1.072)	0.72
Diabetes mellitus	0.917 (0.878-0.958)	<0.001
Hypertensive disease	0.937 (0.897-0.979)	0.01
End-stage renal disease	0.802 (0.732-0.878)	<0.001
Coronary heart disease	0.927 (0.879-0.979)	0.01
Cerebrovascular disease	1.130 (1.080-1.182)	<0.001

## Discussion

Our study used a large retrospective cohort from the NHIS database in South Korea to investigate the association between schizophrenia and PD. The primary findings revealed that individuals with schizophrenia had a higher risk of developing PD than those without schizophrenia. This association remained statistically significant even after adjusting for relevant covariates, with an aHR of 3.198, indicating a strong link between schizophrenia and PD. Among the covariates, head injury, cerebrovascular disease, and neurotic, stress-related, and somatoform disorders were significantly associated with an increased risk of PD. Among the most commonly prescribed antipsychotic drugs, quetiapine was associated with a reduced risk of PD compared with non-users, whereas risperidone was associated with an increased risk.

The strong association between schizophrenia and PD may be explained by shared mechanisms between these disorders, particularly dopamine dysregulation. Schizophrenia is characterized by excessive mesolimbic dopamine transmission, whereas PD is marked by progressive dopamine neuron loss in the substantia nigra. These opposing pathways may increase the vulnerability of schizophrenia patients to PD. Beyond dopamine dysregulation, alterations in dopamine receptor signaling, mitochondrial dysfunction, neuroinflammation, abnormal synaptic plasticity, and gut–brain axis disturbances further contribute to this susceptibility. Dysregulation of D2-like receptors may exacerbate neurodegeneration when schizophrenia patients develop PD [39]. Both disorders also exhibit Complex I dysfunction in the electron transport chain, which leads to oxidative stress and neuronal damage [40]. Chronic neuroinflammation and excessive microglial activity may further accelerate neurodegeneration, and are potentially influenced by gut–brain axis [41].

Head injury may elevate PD risk through mechanisms involving axonal injury and impaired transport, which influence the formation of pathological proteins such as amyloid-β peptides and hyperphosphorylated tau [42]. Notably, amyloid precursor protein (APP), α-synuclein (α-syn), hyperphosphorylated tau, and TAR DNA-binding protein 43 (TDP-43) are frequently reported after traumatic brain injury (TBI) and are closely linked to PD [43]. Additionally, extracellular glutamate accumulation and olfactory dysfunction are pathological conditions shared by both TBI and PD, suggesting common pathological molecular mechanisms, which may contribute to the increased PD risk following TBI [44].

Cerebrovascular disease may increase the risk of PD in schizophrenia patients through several mechanisms. Ischemic stroke induces PD-like pathology, including α-synuclein aggregation and dopaminergic neuron loss, which may exacerbate neurodegeneration [45]. Neuroinflammation triggered by cerebrovascular disease may accelerate neurodegenerative processes, while shared pathogenic mechanisms involving abnormal protein accumulation further increase PD susceptibility in schizophrenia patients. Supporting these hypothesis, another study demonstrated that cerebrovascular risk factors, including prior stroke, are significantly associated with an increased risk of PD. These factors can contribute to strategic infarcts and white matter lesions, which may exacerbate neurodegeneration in vulnerable populations, such as patients with schizophrenia [34].

Neurotic, stress-related, and somatoform disorders, including anxiety and obsessive-compulsive disorders, were also associated with an increased risk of PD. According to Lin et al., anxiety disorders significantly increase the likelihood of developing PD, with the severity of anxiety correlating with a higher risk [46]. Similarly, previous study found that anxiety and depression may precede PD onset as prodromal non-motor symptoms, suggesting shared pathophysiological mechanisms possibly involving neurotransmitter dysregulation and neuroinflammation [47]. These findings indicate that neurotic and stress-related disorders may amplify neurodegenerative processes, contributing to the increased risk of PD observed in schizophrenia patients.

We found a weak positive association between the overall N05A drug exposure and an increased risk of PD. Antipsychotic drugs block D2 receptors, thereby increasing the activity of indirect pathway medium spiny neurons (MSNs) that express D2 receptors. Based on this mechanism, the overactivation of indirect pathway MSNs is thought to suppress motor function, potentially contributing to an increased risk of Parkinson’s disease (PD) [48]. Notably, quetiapine, risperidone, and aripiprazole were the three most commonly prescribed N05A drugs in our cohort, yet they demonstrated divergent associations with PD risk. This contrast likely contributed to the weak overall association. Quetiapine has a higher affinity for serotonin 5-HT2 receptors than for dopamine D1 and D2 receptors [49–51]. Conversely, risperidone exhibits a higher dopamine receptor occupancy rate than quetiapine, exerting a stronger influence on dopaminergic pathways [52–53]. Aripiprazole differs mechanistically as a high-affinity D2 partial agonist, and molecular imaging studies indicate that D2/3 receptor occupancy can be very high at clinically effective doses. [54–55] Although partial agonism may reduce net dopaminergic blockade compared with pure antagonists, very high receptor engagement could still contribute to parkinsonism-related outcomes in susceptible individuals. This differential receptor affinity may underlie the contrasting PD risks observed among users of these drugs. In drug-induced parkinsonism, symptoms typically improve after discontinuation of the causative medication, but degenerative PD may still develop even after drug withdrawal [56]. Previous studies have reported that drug-induced parkinsonism can occur from several days to a few months after exposure to antipsychotic medications [57–60]. To minimize the potential misclassification of transient, drug-related parkinsonian symptoms as idiopathic PD, our study defined Parkinson’s disease as new diagnoses made at least one year after the initial schizophrenia diagnosis, which generally coincided with the initiation of antipsychotic treatment. This one-year lag period was applied as a conservative approach to reduce the possibility that early, reversible medication-induced parkinsonism was misclassified as true degenerative PD. This finding indicates that prolonged exposure to neuroleptics may have neurotoxic effects on dopaminergic neurons through mechanisms such as mitochondrial respiratory chain inhibition, increased dopamine turnover, and elevated production of neurotoxic reactive oxygen species [61].

Patients with schizophrenia have been reported to have higher mortality rates than the general population [62–63]. To account for the potential impact of premature death before PD onset, we conducted a competing risk analysis. The results were consistent with those of the standard analysis without competing risks, suggesting that schizophrenia independently increases the risk of PD regardless of mortality. These findings reinforce the robustness of our results.

The limitations of this study include the absence of laboratory test results and the lack of a multicenter design, which may restrict the generalizability of the findings. In addition, the retrospective observational design limits causal inference; therefore, the observed findings should be interpreted as associations rather than definitive evidence of causality. Misclassification of diagnoses based on administrative codes is also a potential concern; however, to improve diagnostic accuracy, we defined diseases as requiring at least one inpatient record or two outpatient records within a year. Furthermore, as this study relies on administrative claims data, access to granular clinical information—such as disease severity, symptom onset timing, and neuroimaging results (e.g., DaTscan)—was restricted. These factors are highly relevant to PD risk and differential diagnosis, and their absence may limit the depth of our clinical interpretation. In particular, reflecting individual daily dosing ranges or the exact cumulative duration of exposure was challenging within the current analytical framework of this large-scale administrative database. The lack of such dose-response data may limit the pharmacological interpretation of the association between specific medications and PD risk.

As the analysis focused on drugs with larger sample sizes, individual analyses for all medications were not performed. While multiple comparisons across different antipsychotic agents were not explicitly adjusted, which may increase the risk of Type I error, we addressed potential confounding by incorporating a wide range of clinical and demographic covariates into our multivariate models. Additionally, medication adherence could not be directly assessed, but the intent-to-treat approach was applied to mitigate this limitation.

Furthermore, although collinearity analysis was conducted to reduce confounding effects by excluding highly correlated variables, residual bias may persist, particularly due to the close association between schizophrenia diagnosis and antipsychotic medication use (confounding by indication). Moreover, we could not fully account for time-varying treatment patterns, such as medication switching or the use of anticholinergics, which may underlie the potential for misclassifying persistent medication-induced symptoms as incident PD. In addition, despite the 1-year lag period, the risk of reverse causation cannot be entirely dismissed given PD’s long prodromal phase, particularly regarding the complex temporal relationship between schizophrenia onset, medication exposure, and PD diagnosis. Although the clinical rarity of overt psychosis as an early feature likely limits this bias [64–65]. These potential biases should be considered when interpreting the study results.

Despite these limitations, our study has several strengths. Compared to previous studies, very few have investigated the association between schizophrenia and Parkinson’s disease in Asian populations. Unlike prior studies that broadly analyzed psychiatric disorders or did not sufficiently adjust for comorbidities and medication use, we specifically focused on schizophrenia patients, included a wide range of covariates, and utilized a cohort design with competing risk adjustment. Additionally, we performed a focused comparison of two antipsychotic medications to explore their differential impacts on PD risk.

## Conclusions

This study is the first to evaluate the association of schizophrenia and PD using a large claims data including majority of Korean population. The findings highlight the need for additional research to explore the underlying mechanisms and broader implications of this association. Further research is needed to investigate the dose–response relationship between antipsychotic use and the risk of PD in schizophrenia patients.

## Supporting information

S1 Fig The frequency of prescribe ATC codes at the first schizophrenia diagnosis. (TIF)

S2 Fig The frequency of medications prescribed for the first schizophrenia diagnosis. (TIF)
