# Supplementary figures and images for: Risk of Parkinson’s disease in patients with schizophrenia: Impact of antipsychotic medication use
Source: PLoS One. 2026 May 4;21(5):e0346233. doi: 10.1371/journal.pone.0346233 (PMC13138754; doi:10.1371/journal.pone.0346233)

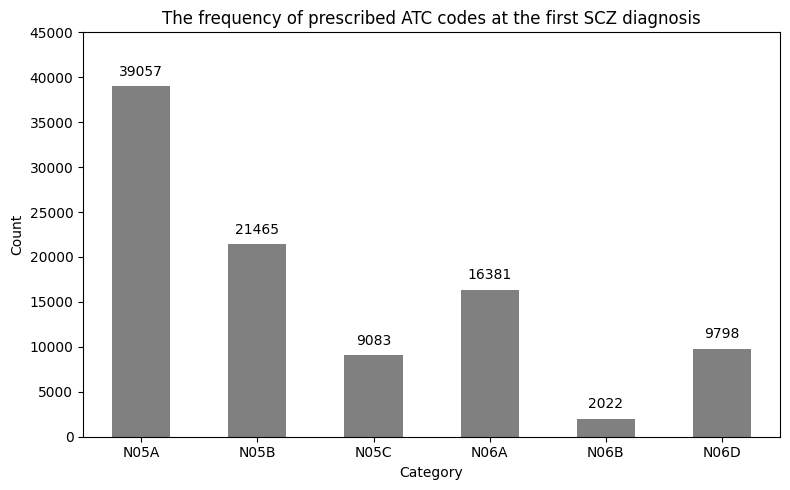

Supplement: S1 Fig — (TIF) [file pone.0346233.s001.tif]

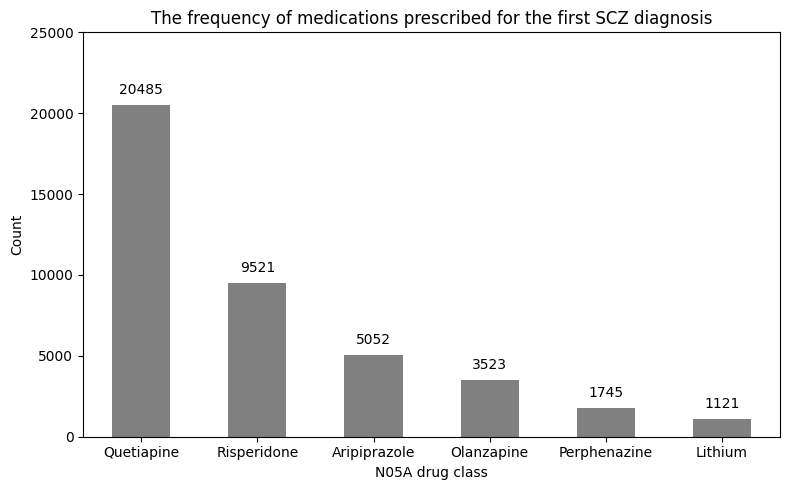

Supplement: S2 Fig — (TIF) [file pone.0346233.s002.tif]
